# Unmasking Non-small Cell Lung Cancer: The Unusual Trail of Hip Pain and Skeletal Metastasis

**DOI:** 10.7759/cureus.39404

**Published:** 2023-05-23

**Authors:** Hamad Ahmad, Hoore Jannat, Urooj Khan, Noaman Ahmad

**Affiliations:** 1 Internal Medicine, Westchester Medical Center, Valhalla, USA; 2 Internal Medicine, Khyber Medical College, Peshawar, PAK; 3 Internal Medicine, Khyber Medical University, Peshawar , PAK; 4 Internal Medicine, Huntsville Hospital, Huntsville, USA

**Keywords:** hip joint pain, multidisciplinary approach, pelvis reconstruction, lung adenocarcinoma, metastatic carcinoma

## Abstract

We present a case of a 68-year-old female with a history of hypertension and hypothyroidism who presented to the emergency department with right lower extremity pain and difficulty ambulating. An initial evaluation revealed an abnormal appearance of the right hip on MRI, concerning avascular necrosis versus acetabular compression fracture. Subsequent diagnostic procedures, including joint aspiration and radiologic bone biopsy, led to the surprising discovery of metastatic lung carcinoma on tissue pathology. Further work-up reveals lung primary adenocarcinoma with additional metastases to the brain as well. The patient underwent resection of acetabulum and complex surgical pelvis reconstruction, irradiation for brain metastases, and rehabilitation. This case highlights the importance of considering atypical presentations of metastatic malignancies and the need for a multidisciplinary approach to optimize patient management.

## Introduction

Non-small cell lung cancer (NSCLC) is the most common type of lung cancer, accounting for over 85% of all occurrences [1]. It is a diverse group of tumors that vary from small-cell lung cancer in histology, therapy, and overall prognosis. Because the primary tumor in NSCLC starts in the lungs, symptoms connected to the respiratory system are common. A persistent cough, shortness of breath, chest pain, wheezing, and recurring respiratory infections are the most prevalent symptoms. Weight loss, weariness, loss of appetite, and hoarseness of voice may also be the presenting symptoms [2]. Furthermore, some people may experience symptoms associated with the involvement of other organs, such as bone pain, neurological impairments, or indications of distant metastases. Early-stage NSCLC is typically asymptomatic, which delays diagnosis and detection in later stages. Metastatic NSCLC frequently affects distant organs such as the liver, bones, brain, and adrenal glands. Skeletal metastases affect 30-40% of patients with advanced NSCLC [3]. As a result, it is critical to be aware of both common and uncommon NSCLC appearances. This post will go over the uncommon incidence of hip pain as a presenting sign of metastatic NSCLC.

## Case presentation

**Figure 1:**
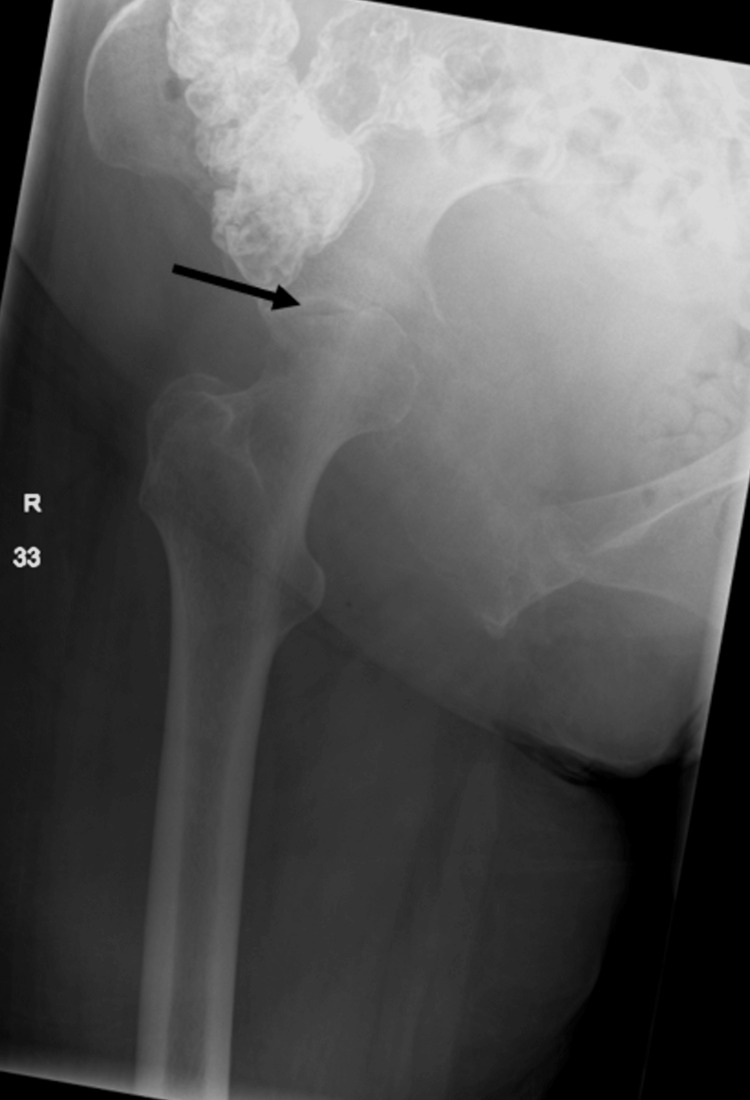
X-ray of right hip joint showing age-indeterminate deformity (arrow); may represent compression fracture vs. avascular necrosis

A 68-year-old female with a history of hypertension and hypothyroidism presented with right hip discomfort for three months and difficulty walking for one week due to worsening pain. The patient went through multiple sessions of outpatient physical therapy with little improvement in her symptoms. The patient was afebrile when she arrived and had normal blood testing, including a normal leukocyte count and inflammatory markers. The right hip x-ray demonstrated an age-indeterminate deformity of compression fracture vs. avascular necrosis with concomitant osteophytes and osteopenia (Figure 1).

**Figure 2:**
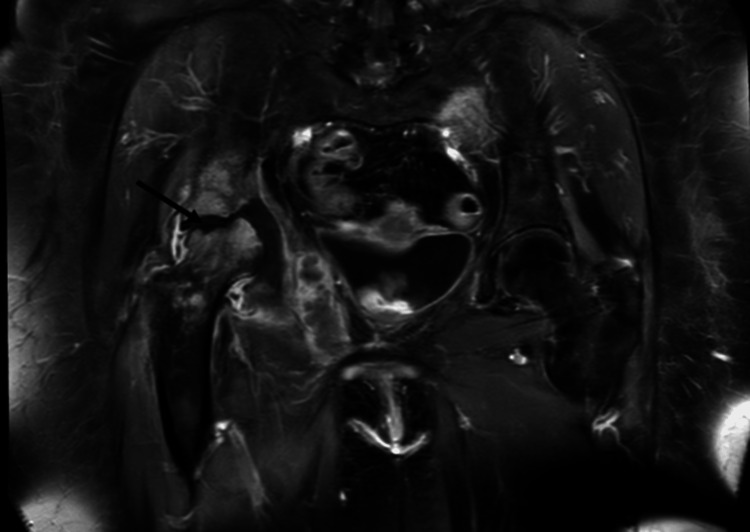
MRI of pelvis showing destroyed acetabular head (arrow)

**Figure 3:**
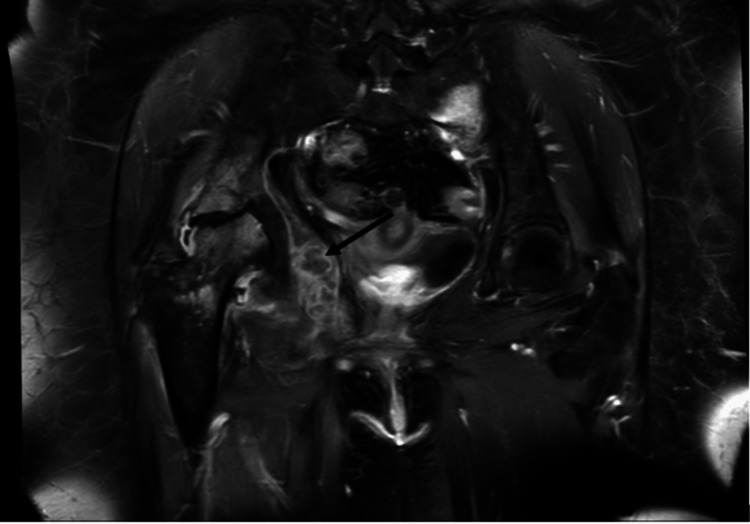
MRI of pelvis showing the peripherally-enhanced fluid collection (arrow) in the right obturator internus muscle measuring 3.7 x 2.1 x 3.9 cm, which may represent an abscess

A magnetic resonance imaging (MRI) scan of the pelvis revealed likely osteomyelitis or septic arthritis with intramuscular abscesses (Figures 2-3).

**Figure 4:**
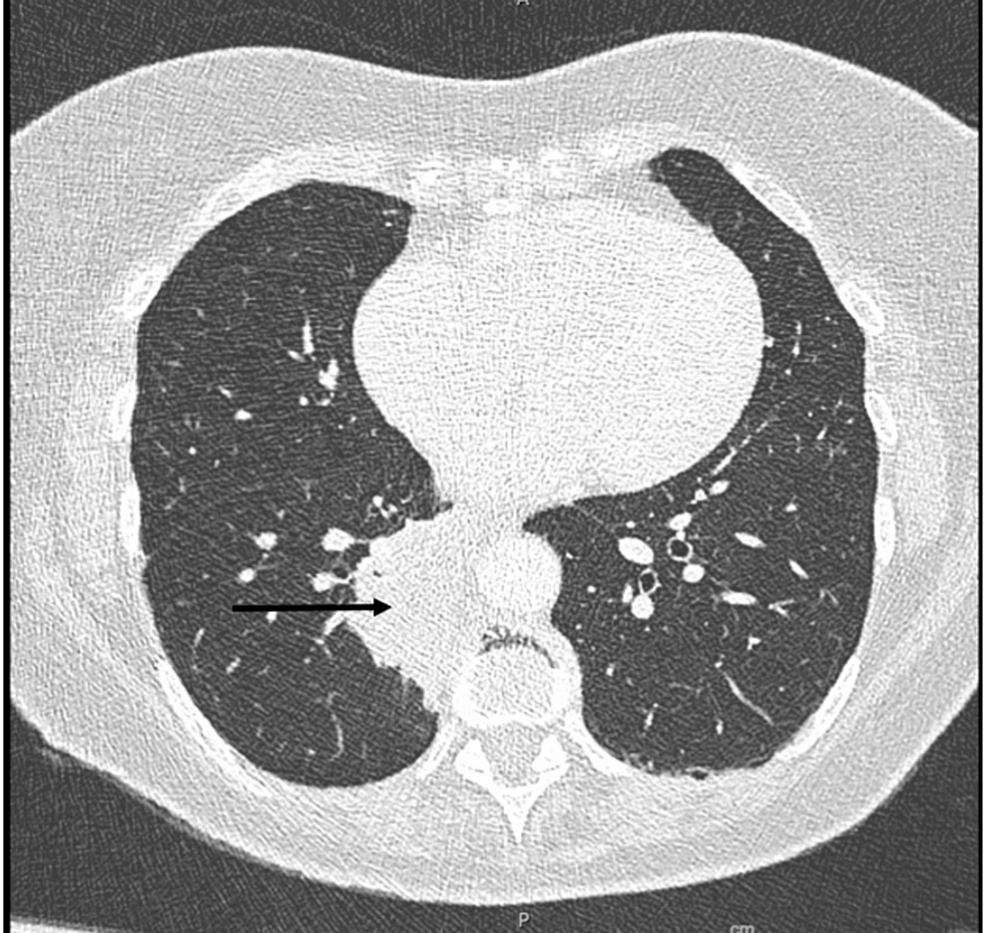
CT of chest showing a 4.1 x 2.4 x 2.6 cm heterogenous mass (arrow) in the right inferior perihilar region with associated pleural reaction and application to the T10 vertebral body

A right hip joint radiologic aspiration with biopsy was done, and 4 cc of purulent fluid and bone samples were collected and sent for culture and tissue pathology. Because the imaging findings suggested right pelvic osteomyelitis or septic arthritis, the patient was started on empiric vancomycin and ceftriaxone. The joint aspiration cultures and blood cultures, however, came out negative, but tissue pathology showed metastatic lung cancer. Antibiotics were stopped, and an oncology workup was initiated, which included a CT scan of the chest to find the underlying lung carcinoma, a CT abdomen/pelvis with IV contrast, a brain MRI, and a nuclear bone scan to screen for metastatic disease elsewhere. A worrisome right perihilar malignancy, indicating bronchogenic cancer, was discovered on a chest CT scan, as well as metastatic lymphadenopathy in the right pulmonary hilar region (Figure 4).

**Figure 5:**
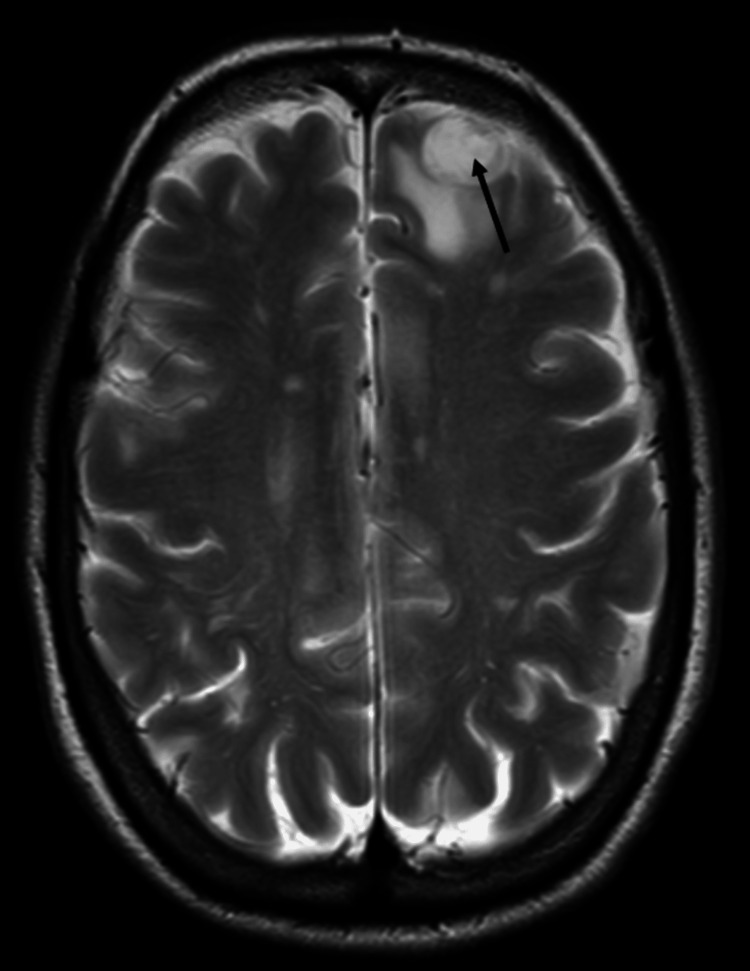
MRI of the brain showing the single 15x13 mm metastasis (arrow) with surrounding vasogenic edema in the left anterior frontal lobe

On the nuclear bone scan, increased radiotracer uptake was seen in the right ischium, left sacroiliac joint, and perhaps in the T12 pedicle. Furthermore, the MRI of the brain revealed three distinct metastases in the left frontal lobe and cerebellum (Figure 5).

Interventional radiology, orthopedics, medical oncology, radiation oncology, and neurosurgery evaluated the patient during a multidisciplinary meeting and determined that the patient should undergo resection of the affected acetabulum and complex surgical pelvic reconstruction with preoperative embolization of the tumor-feeding arteries, have stereotactic radiosurgery for the brain metastases, and immunotherapy for the primary lung cancer. The patient tolerated all procedures well, was discharged to a rehabilitation facility, and is presently undergoing immunotherapy as an outpatient.

## Discussion

Lung cancer is the leading cause of cancer-related deaths worldwide. About 2.2 million people are diagnosed with lung cancer every year, and 1.8 million of these die [1]. In the United States, an estimated 235,000 patients are diagnosed with lung cancer each year, resulting in over 130,000 deaths [4]. The majority of lung cancer patients present with advanced disease, possibly due to the aggressive nature of the illness and the absence of noticeable symptoms until the disease has progressed locally or metastasized. Histologically, approximately 95% of lung cancers are classified as either small-cell lung cancer or NSCLC. This distinction is crucial for accurate staging, treatment, and prognosis, as the two types exhibit significant differences in biological behavior, natural progression, and response to therapy.

The most common symptoms upon presentation include cough, dyspnea, pain, and weight loss. Symptoms of lung cancer can arise from the tumor's local effects, regional or distant spread, or secondary to paraneoplastic syndromes [2]. Bone is one of the most common and earliest sites of metastases from lung cancer [5]. Bone metastases typically present with pain in the back, chest, or extremities, along with elevated levels of serum alkaline phosphatase. Not uncommonly, it also presents with a pathological fracture, spinal instability, spinal cord compression, and hypercalcemia. Our patient, being unique, did not have any respiratory symptoms, and surprisingly, despite metastasis to the bone, she did not have elevated alkaline phosphatase or hypercalcemia. The vertebral bodies, ribs, pelvis, and proximal long bones are the areas most frequently affected. Bone metastases usually occur at an advanced stage and adversely affect the patient's quality of life, but they can present as an initial symptom [5], as in our patient. Other common sites of metastasis include the brain, and the likelihood of brain metastasis increases with larger primary tumor sizes and the presence of regional lymph node involvement [6]. Brain metastasis symptomatology depends on the site of the metastasis. Our patient, though she had metastases in the frontal lobe, cerebellum, and semioval, was completely asymptomatic.

All patients with suspected lung cancer should have contrast-enhanced chest computed tomography [7]. Surgical resection provides the best chance for long-term survival and cure in patients with early-stage NSCLC. Stage I or II NSCLC patients should undergo complete surgical resection whenever possible [8]. Our patient, who was already at stage 4, was not a candidate for resection. For those with pathologically confirmed stage III disease, a combined modality approach using concurrent chemoradiotherapy is typically preferred, then immunotherapy if there is no progression [9]. Patients with stage IV disease are typically treated with palliative systemic therapy, immunotherapy, or a symptom-focused palliative approach [10], as in our patient. In cases where there is an isolated metastasis (e.g., brain or adrenal gland) indicating stage IV disease, patients may benefit from radiation therapy or resection of the metastasis, along with aggressive treatment of the primary tumor [11]. Our patient responded well to radiotherapy, and repeat MRI brain scans showed decreased size and configuration of previously described lesions in the left frontal lobe, right centrum semiovale, and right medial cerebellar hemisphere.

In NSCLC, genotype subtype analysis and the development of targeted therapies for specific gene mutations have led to personalized treatment approaches. Targeted therapy has demonstrated superior responses compared to standard chemotherapy. Our patient received two cycles of chemotherapy with pemetrexed and carboplatin. Currently, specific subsets of NSCLC with mutations in the epidermal growth factor receptor (EGFR), B-Raf proto-oncogene (BRAF), echinoderm microtubule-associated protein-like 4-anaplastic lymphoma kinase (EML4-ALK) fusion oncogene, program death ligand-1 (PD-L1), and ROS proto-oncogene 1 (ROS1) fusions have established targeted therapies as standard treatment [12]. Our patient is currently on nivolumab and ipilimumab and is tolerating them well.

The most influential factor affecting prognosis in patients with NSCLC is the tumor, node, and metastasis (TNM) stage at the time of diagnosis. However, there are other clinical factors that can independently predict survival, regardless of the disease stage. Poor performance status, indicating a patient's overall health and ability to perform daily activities, is associated with a less favorable prognosis [13]. Additionally, a low degree of tumor differentiation, indicating the extent to which cancer cells resemble normal cells, is linked to poorer outcomes [14]. Moreover, the absence of specific mutations that can be targets for immunotherapy is also associated with a less favorable prognosis [15]. These factors beyond the TNM stage contribute to the overall assessment of prognosis in NSCLC patients. Being diagnosed at an advanced stage and having poorly differentiated carcinoma on pathology makes her prognosis very poor.

## Conclusions

NSCLC typically presents with respiratory symptoms but, in rare cases, it can manifest atypically. Bone metastasis is a common sequela of advanced lung cancer, but it is very uncommon to be the first presenting symptom of this devastating disease. Our patient presented with hip pain due to skeletal metastasis without any respiratory or systemic symptoms. Hence, it is important to be vigilant and consider a metastatic process as one of the differentials for a very common symptom like hip pain, which will help in early diagnosis, prompt appropriate management, and help prevent the further spread of the disease.
